# Substitution of manure for chemical fertilizer affects soil microbial community diversity, structure and function in greenhouse vegetable production systems

**DOI:** 10.1371/journal.pone.0214041

**Published:** 2020-02-21

**Authors:** Haoan Luan, Wei Gao, Shaowen Huang, Jiwei Tang, Mingyue Li, Huaizhi Zhang, Xinping Chen, Dainius Masiliūnas

**Affiliations:** 1 Key Laboratory of Plant Nutrition and Fertilizer, Ministry of Agriculture/Institute of Agricultural Resources and Regional Planning, Chinese Academy of Agricultural Sciences, Beijing, China; 2 Center for Resources, Environment and Food Security, China Agricultural University, Beijing, China; 3 Tianjin Institute of Agricultural Resources and Environment, Tianjin, China; 4 Laboratory of Geo-information Science and Remote Sensing, Wageningen University, Wageningen, The Netherlands; RMIT University, AUSTRALIA

## Abstract

Soil microbial communities and enzyme activities together affect various ecosystem functions of soils. Fertilization, an important agricultural management practice, is known to modify soil microbial characteristics; however, inconsistent results have been reported. The aim of this research was to make a comparative study of the effects of different nitrogen (N) fertilizer rates and types (organic and inorganic) on soil physicochemical properties, enzyme activities and microbial attributes in a greenhouse vegetable production (GVP) system of Tianjin, China. Results showed that manure substitution of chemical fertilizer, especially at a higher substitution rate, improved soil physicochemical properties (higher soil organic C (SOC) and nutrient (available N and P) contents; lower bulk densities), promoted microbial growth (higher total phospholipid fatty acids and microbial biomass C contents) and activity (higher soil hydrolase activities). Manure application induced a higher fungi/bacteria ratio due to a lower response in bacterial than fungal growth. Also, manure application greatly increased bacterial stress indices, as well as microbial communities and functional diversity. The principal component analysis showed that the impact of manure on microbial communities and enzyme activities were more significant than those of chemical fertilizer. Furthermore, redundancy analysis indicated that SOC and total N strongly influenced the microbial composition, while SOC and ammonium-N strongly influenced the microbial activity. In conclusion, manure substitution of inorganic fertilizer, especially at a higher substitution rate, was more efficient for improving soil quality and biological functions.

## 1. Introduction

Soil, a dynamic living system, provides various ecosystem services including nutrient cycling, carbon (C) sequestration, and water regulation [1]. Microorganisms are one of the important parts of soil and play an important role to mediate many processes, including soil aggregate formation [2], soil organic C (SOC) decomposition and nutrient transformation [3]; meanwhile, the variations in the abundance and composition of soil microbial community are critical indicators of soil biochemical processes and crop productivity in agricultural systems [4]. Soil extracellular enzymes, produced and secreted by soil microorganisms, provide a functional fingerprint of microbial communities because they are often involved in SOC formation and nutrient cycling [5]. Additionally, soil microbial community and extracellular enzymes could be influenced by edaphic, climatic, and other characteristics; however, which factors become the main determinant depends on the ecosystem types and environmental conditions [6]. Thus, the mechanisms of controlling soil microbial characteristics in several special agroecosystems (e.g., vegetable production systems) need further research.

Fertilization, a common agricultural management practice, has been found to affect soil microbial biomass, activity and community composition in agricultural production systems [7], through the modification of soil physicochemical properties. For example, several studies reported that organic fertilization (e.g., manure and straw) had positive effects on soil microbial biomass and activity [8, 9]; however, chemical fertilization was not beneficial for these microbial variables [8]. All those studies were mostly used to investigate the effects of different fertilization treatments (chemical vs organic fertilization) on soil microbial characteristics in open-air agricultural fields. However, little information is available on the response of soil microbial characteristics to the application of different fertilization treatments in vegetable fields.

Vegetable production is a vital part of the agricultural sector in the world. As the largest producer and consumer of vegetables, the area of vegetable planting in China was 2.1 × 10^7^ ha [10] in 2015, which was around 9.5 and 210.0 times, respectively, of that in Europe (2.2 × 10^6^ ha [11]) and Canada (1.0 × 10^5^ ha [12]). Over the last 30 years, greenhouse vegetable production (GVP) systems in China have grown rapidly and have become the main type of vegetable production due to its higher economic benefits relative to vegetable production in open-air fields [13]. However, to obtain high yields within short periods, growers often use large amounts of fertilizers, especially nitrogen (N) fertilizers. It was reported that the rate of N application in GVP systems (up to 1000 kg N ha^−1^) was approximately 7 times that in cropland [14]. However, excess chemical N addition can reduce vegetable quality and simultaneously leads to a series of soil and environmental problems (i.e., soil acidification and biological quality degradation) in the GVP system [15]. Moreover, as a high-intensity agricultural ecosystem, GVP systems are characterized by its closed or semi-closed environment with high inside temperature and humidity, which has led to a series of ecological and biological problems in soils (e.g., soil acidification, salinization, and biodiversity degradation) [16, 17]; in turn, these problems had adverse effects on the sustainability of agricultural production. For solving these problems, organic amendments and substitution of chemical fertilizers are increasingly recommended as effective and sustainable practices to sustain crop yield and soil quality [18, 19]. Organic fertilizers like manure can effectively increase microbial biomass and activity in the soil, and modify soil microbial communities [20]. Nevertheless, the effects of manure application on soil microorganisms vary with soil type, climate, and quantity and quality of manure [8, 21]. Thus, the impact of manure application on soil microbial properties and the underlying mechanisms still need to be investigated in GVP systems.

The objectives of this study were (1) to investigate the effects of 8 years of continuous manure application on soil physicochemical properties (e.g., SOC, available N, and P, etc.), microbial community composition and extracellular enzyme activities, and (2) to identify the main controlling environmental factors that drive the changes in soil enzyme activities and microbial community compositions in GVP systems. We predicted that manure application would improve soil physicochemical properties and increase soil microbial biomass and activity and that these positive effects of manure application would be enhanced with increasing manure addition rate.

## 2. Materials and methods

### 2.1 Study site

An 8-year field experiment with celery (*Apium graveolens cv*. *Wentula*) -tomato (*Lycopersicon esculentum cv*. *Chaoyan No*. *299*) rotation system was started in October 2009 at a solar greenhouse farm in Xiqing District, Tianjin City, China (117°0′E, 39°13′N). This region has a warm sub-humid continental climate. The mean annual temperature is 11.6°C with mean annual precipitation of 586 mm. The frost-free period is 203 days and the annual sunshine duration is 2810 h. The soils in the experimental site are medium-loam Chao (Aquic cambisols) soil (FAO classification) with the groundwater depth of 100 cm. Some of the initial physicochemical characteristics of the surface (0–20 cm) soil were shown in Table 1.

**Table 1:** Characteristics of the soil sampled in 2009.

Parameters	pH	SOC (g kg^−1^)	NO_3_^−^-N (mg kg^−1^)	Available P (mg kg^−1^)	Available K (mg kg^−1^)
Soil samples (2009)	7.9	15.3	186.2	144.6	404.0

SOC soil organic carbon, NO_3_^−^-N nitrate nitrogen, P phosphorus, K potassium.

### 2.2. Experimental design

This study comprised five treatments as follows: (1) no fertilizer nitrogen (N) inputs (0N), (2) 100% inorganic N (IN) inputs (100IN), (3) 75% IN and 25% organic N (ON) inputs (75IN/25ON), (4) 50% IN and 50% ON inputs (50IN/50ON), and (5) 25% IN and 75% ON inputs (25IN/75ON). All treatments except 0N received 450.0 (N), 225.0 (P_2_O_5_) and 600.0 (K_2_O) kg ha^–1^ in the tomato season, and 450.0 (N), 300.0 (P_2_O_5_) and 600.0 (K_2_O) kg ha^–1^ in the celery season. A detailed description of the N and C inputs is shown in Table 2.

**Table 2:** Manure, nitrogen and carbon inputs (kg ha^−1^) used in each treatment during the spring tomato season and autumn-winter celery season.

Treatments	Manure inputs	N inputs			C inputs
		Chemical fertilizer	Pig manure	Total	Pig manure
Spring tomato season					
0N	0	0	0	0	0
100IN	0	450.0	0	450.0	0
75IN/25ON	5183.0	337.5	112.5	450.0	1130.0
50IN/50ON	10367.0	225.0	225.0	450.0	2260.0
25IN/75ON	15550.0	112.5	337.5	450.0	3390.0
Autumn-winter celery season					
0N	0	0	0	0	0
100IN	0	450.0	0	450.0	0
75IN/25ON	5183.0	337.5	112.5	450.0	1130.0
50IN/50ON	10367.0	225.0	225.0	450.0	2260.0
25IN/75ON	15550.0	112.5	337.5	450.0	3390.0

The inorganic fertilizers were urea (N 46%), calcium superphosphate (P_2_O_5_ 12%), diammonium phosphate (N 18%, P_2_O_5_ 46%), potassium chloride (K_2_O 60%) and monopotassium phosphate (P_2_O_5_ 52%, K_2_O 34%). The commercial pig manure (i.e., organic manure) produced by Jiangsu Tianniang Agricultural Science & Technology Co., Ltd, had a C content of 218.0 g kg^−1^, N content of 21.7 g kg^−1^, P_2_O_5_ content of 13.9 g kg^−1^, K content of 16.3 g kg^−1^ (by dry weight) and its water content was 28.9%. More importantly, these pig manures were sterilized and have little microorganisms (S2 Table).

Manure was applied as basal fertilization. During the tomato season, chemical fertilizers (20% N, 70% P_2_O_5_, and 20% K_2_O) were applied as basal fertilization. The remaining N and K_2_O were applied as top dressings in four doses (30% N and 10% K_2_O in the flowering period, then 30% N and 30% K_2_O, 10% N and 30% K_2_O, and 10% N and 10% K_2_O in the first, second, and third fruit cluster expansion periods, respectively). The remaining P_2_O_5_ was applied as top dressing in two doses (15% P_2_O_5_ in the flowering period and 15% P_2_O_5_ in the first fruit cluster expansion period). During the celery season, the same amount of chemical fertilizer as tomato season was applied as basal fertilization. The remaining N and K_2_O were applied as top dressings in three doses (35% N and 10% K_2_O, 35% N and 35% K_2_O, and 10% N and 35% K_2_O in the 5–6 leaf, 8–9 leaf, and 11–12 leaf periods, respectively). The remaining P_2_O_5_ (30%) was applied as a top dressing in the 5–6 leaf period. The basal fertilizer was broadcast and then tilled into the soil using a rotary tilling device, and the top dressing fertilizers were dissolved in water and then poured onto the soil.

All treatments were laid out in a randomized block design with three replicates. Each plot was 14.4 m^2^ (2.4 m × 6.0 m), and neighboring plots were separated with PVC plates 105 cm deep (100 cm underground, 5 cm aboveground) to avoid nutrients and water being transferred between neighboring plots. The tomato planting density was 25000 plants ha^−1^, with rows 0.3 m apart and plants 0.6 m apart. The celery planting density was 330570 plants ha^−1^, with rows 0.15 m apart and plants 0.2 m apart. Each plot was fitted with a water meter to ensure the amount of irrigation applied was accurately controlled. The total irrigation amounts during the celery and tomato growing periods were 3334 and 3889 m^3^ ha^–1^ year^–1^, respectively.

### 2.3 Soil sampling

After the celery harvest in January 2017, ten soil cores (3 cm in diameter, 0–20 cm depth) were collected per plot and mixed to create one combined soil sample. The fresh soil samples were sieved through a 2-mm mesh and divided into two subsamples. One subsample was air-dried and then passed through 0.25 mm or 2 mm mesh for the determination of soil physicochemical properties (0.25 mm for soil organic carbon and total nitrogen; 2 mm for soil available nutrients), the other subsample was stored at 4°C (one day) for the measurement of soil extracellular enzyme activities, soil microbial biomass, and community composition.

### 2.4 Soil physicochemical analysis

Soil pH was determined with a compound electrode (PE 10, Sartorius, Goettingen, Germany) using a soil/water ratio of 1:2.5(w/v; g cm^-3^). Electrical conductivity (EC) was measured at 25°C in 1:5 soil-water mixtures. Soil bulk density was measured using the cutting ring method after drying the soil cores at 105°C for 48 h. Soil organic carbon (SOC) and total nitrogen (TN) were determined by the heated dichromate/titration method [22] and the Semimicro-Kjeldahl method [23], respectively. Soil nitrate-N (NO_3_^−^N) and ammonium-N (NH_4_^+^-N) was extracted by 2 M potassium chloride (KCl; soil/KCl ratio of 1:5) and measured using a flow injection autoanalyzer (Smartchem 200, Alliance, France). Soil available phosphorus (P) was extracted by 0.5 M sodium bicarbonate (pH 8.5) and determined by the Olsen method [24]. Soil available potassium (K) was extracted by 1 M ammonium acetate, adjusted to pH 7.0, and then measured by atomic absorption spectrometry (NovAA300, Analytik Jena AG). Soil microbial biomass carbon/nitrogen (MBC/MBN) was measured by the chloroform fumigation-extraction method and determined by C/N analyzer (Multi N/C 3100/HT1300, Analytik Jena AG, Germany) [25]. Moreover, the values of MBC and SOC were used to calculate the microbial quotient (MQ, the ratio of MBC to SOC), which could be used as indicators of soil microbial activity [26].

### 2.5 Phospholipid fatty acid analysis

Soil microbial community composition was determined by phospholipid fatty acid (PLFA) analysis according to the procedure described by DeForest [27]. Briefly, the soil samples were freeze-dried at –80°C, then PLFAs were extracted using a 1:2:0.8 (by volume) chloroform–methanol–citrate buffer mixture at pH 4.0. The chloroform extract was passed through an SPE-Si column (Supelco, Poole, UK), then the neutral lipids, glycolipids, and polar lipids were eluted with chloroform, acetone, and methanol, respectively. Nonadecanoic acid methyl ester (19:0) was added as an internal standard, and the recovered polar lipids were converted into fatty acid methyl esters (FAMEs) by a mild alkaline methanolysis. Dried FAMES were redissolved in *n*-hexane and then quantified by gas chromatography (N6890, Agilent Technologies, Santa Clara, CA, USA) and identified with a MIDI Sherlock microbial identification system version 4.5 (MIDI Inc., Newark, DE, USA). The total and individual PLFA abundances were expressed in units of nmol g^−1^ soil. The PLFAs were divided into various taxonomic groups based on previously published PLFA biomarker data [9, 28]. Specifically, 15:00, i15:0, a15:0, i16:0, 16:1ω7c, 17:00, a17:0, i17:0, cy17:0, 18:1ω7c and cy19:0 were used to represent bacterial biomarkers [29]. The PLFA i15:0, a15:0, i16:0, a17:0 and i17:0 were used as biomarkers for Gram-positive (G+) bacteria, and the PLFA 16:1ω7c, cy17:0, 18:1ω7c and cy19:0 were designated for Gram-negative (G−) bacteria [30]. The general PLFAs (15:00 and 17:00) were summed as indicators of other bacteria [28]. The PLFAs 18:2ω6c and 18:1ω9c were attributed to saprotrophic fungi (SF), whereas PLFAs 16:1ω5c were regarded as arbuscular mycorrhizal fungi (AMF) [31]. The PLFAs 10Me-16:0, 10Me-17:0 and 10Me-18:0 were calculated as indicators of actinomycete [3]. Other PLFAs such as 14:00, 16:00 and 18:00 were also used to calculate the total PLFAs [28]. The total PLFAs were calculated by summing all of the PLFAs mentioned above and used as an index for total microbial biomass. Ratios of group-specific lipids (i.e., F/B, G+/G− and AMF/SF) were taken to reflect the relative biomass of their respective groups [32]. The ratios of cyclopropyl fatty acids to their precursors (cy17:0/16:1ω7c) and (i17:0 + i15:0) to (a17:0 + a15:0) as two bacterial physiological stress indices were evaluated in soil microbial community [33].

Moreover, soil microbial community diversity was evaluated using the Shannon–Wiener diversity index (H′_M_), Pielou evenness index (J), and Margalef richness index (SR) based on the following equation [34]:

$${\text{H}\prime }_{\text{M}}=-\Sigma_{\text{i}=1}^{\text{S}}\text{Pi}\times \text{ln}\;\text{Pi},$$

$$\text{J}=\frac{{\text{H}\prime }_{\text{M}}}{{\text{H}\prime }_{\text{Mmax}}};{\;\text{H}\prime }_{\text{Mmax}}=\text{ln}\;\text{S},$$

$$\text{SR}=\frac{\text{S}-1}{\text{ln}\;\text{N}}$$

where ‘Pi’ is the percentage of the peak area of PLFA to the total area of each sample; H’_Mmax_ is the maximum values of H′_M_; ‘S’ is the total number of microbial PLFAs; and ‘N’ is the amount of microbial PLFAs.

### 2.6 Soil enzyme activity analysis

The activity of seven enzymes (Table 3) involved in C and N cycling were determined using microplate fluorometric assay, according to the procedures of DeForest [35]. Briefly, 1.0 g dry-mass-equivalent of fresh soil was homogenized in 100 mL of 50 mM acetate buffer (pH 8.5). For hydrolase analysis, buffer, sample suspension, 10 mM references and 200 μM substrates (4-methylumbelliferone or 7-amino-4-methylocumarin) were dispensed into the wells of a black 96-well microplate. The microplates were covered and incubated at 25°C for 4 h in the dark, then fluorescence was measured using a microplate fluorometer (Scientific Fluoroskan Ascent FL, Thermo) with 365 nm excitation and 450 nm emission filters. Phenol oxidase and peroxidase were measured in a clear 96-well microplate using the substrate of L-3, 4-dihydroxyphenylalanine (L-DOPA). The dispensed volume and the order of buffer, sample suspension, 25 mM L-DOPA, and 0.3% (w/v) H_2_O_2_ were the same as the fluorometric enzymes. The microplates were covered and incubated at 20°C for 20 h in the dark, then the activities were determined by measuring the absorbance at 450 nm using the microplate fluorometer. The enzyme activities were expressed in nmol h^−1^ g^−1^. For each sample, the geometric mean of the assayed enzyme activities (Gmea), hydrolase (GH) and oxidase (GOR) were calculated as:

$$\text{Gmea}=\sqrt[{\text{7}}]{\text{AG}\times \text{BG}\times \text{CBH}\times \text{BX}\times \text{NAG}\times \text{PHO}\times \text{PER}}$$

$$\text{GH}=\sqrt[{\text{5}}]{\text{AG}\times \text{BG}\times \text{CBH}\times \text{BX}\times \text{NAG}}$$

$$\text{GOR}=\sqrt[{\text{2}}]{\text{PHO}\times \text{PER}}$$

**Table 3:** A detailed description of soil extracellular enzymes in this study.

Soil extracellular enzyme	Enzyme commission number	Enzyme function	Abbreviation
α-Glucosidase	3.2.1.20	Hydrolysis of soluble saccharides	AG
β-Glucosidase	3.2.1.21	Hydrolysis of cellulose	BG
β-Cellobiosidase	3.2.1.91	Hydrolysis of cellulose	CBH
β-Xylosidase	3.2.1.37	Hydrolysis of hemicellulose	BX
N-Acetyl-glucosaminidase	3.1.6.1	Hydrolysis of chitooligosaccharides	NAG
Phenol oxidase	1.10.3.2	Oxidation of lignin	PHO
Peroxidase	1.11.1.7	Oxidation of lignin	PER

Moreover, soil enzyme functional diversity was calculated using the activities of seven enzymes using Shannon’s diversity index (H′_E_) as [36]:

$${\text{H}\prime }_{\text{E}}=-\Sigma_{\text{i}=1}^{\text{7}}\text{Pi}\times \text{LnPi}$$

where Pi is the ratio of each enzyme activity to the sum of all enzyme activities.

### 2.7 Data analysis

The characteristics of soil samples from the plots given different fertilization treatments were analyzed using the SPSS 16.0 software (SPSS Inc. Chicago, IL, USA). One-way ANOVA with Duncan tests was performed to test the significance (*P* < 0.05) of measured variables. Differences in soil enzyme activities or microbial community composition were investigated by performing principal component analysis (PCA) using CANOCO 4.5 software (CANOCO, Microcomputer Power Inc., Ithaca, NY, USA). Redundancy analysis (RDA) was performed using CANOCO 4.5 software to illuminate the relationships between soil microbial community, enzyme activities and physicochemical properties in different fertilization treatments. Monte Carlo permutation tests were performed to assess whether soil microbial community composition or enzyme activities were correlated with soil physicochemical properties.

## 3. Results

### 3.1 Changes in soil physicochemical properties

Organic manure application (75IN/25ON, 50IN/50ON and 25IN/75ON) significantly increased soil pH (0.19–0.23) units above the 100IN treatment (Table 4). Soil electrical conductivity (EC) was highest in 100IN treatment, followed by 0N treatment and lowest in manure-amended treatments (Table 4). Higher rates of manure input (50IN/50ON and 25IN/75ON) significantly decreased soil bulk density by 6.40%−8.12% compared with 100IN treatment. The SOC, NO_3_^−^-N and NH_4_^+^-N contents were lowest in the 0N treatment, while these parameters slightly increased under 75IN/25ON treatment, significantly increased under 50IN/50ON and 25IN/75ON treatments by 32.9%−50.8%, 29.9%−40.7%, and 16.0%−16.8%, respectively, compared to 100IN treatment. The available P contents were significantly increased in manure-amended soils by 7.1%−39.9% compared with those in 100IN-amended soils. In contrast, soil available K content was highest in the 0N treatment and decreased by 7.8%−17.2% upon manure addition. Moreover, with increasing manure application rates, the EC, SOC, NO_3_^−^-N, NH_4_^+^-N and available P contents increased, and the bulk density and available K contents decreased.

**Table 4:** Changes in soil physicochemical characteristics under different fertilization treatments after celery harvest in January 2017 (the fifteenth-season). Data are means ± S.E., n = 3. Different lowercase letters indicate significant differences among different treatments at the *P* < 0.05 level.

Treatment	pH	EC (mS cm^–1^)	Bulk density (g cm^–3^)	SOC (g kg^–1^)	NO_3_^−^N (mg kg^–1^)	NH_4_^+^-N (mg kg^–1^)	Available P (mg kg^–1^)	Available K (mg kg^–1^)
0N	8.5 ± 0.0 a	0.44 ± 0.01 b	1.30 ± 0.03 ab	12.8 ± 0.1 e	13.6 ± 3.6 d	5.0 ± 0.5 b	126.5 ± 1.2 c	542.3 ± 35.7 a
100IN	8.3 ± 0.0 b	0.49 ± 0.01 a	1.32 ± 0.04 a	13.3 ± 0.3 d	21.9 ± 2.1 c	5.6 ± 0.2 ab	119.2 ± 8.3 d	532.8 ± 21.3 ab
75IN/25ON	8.5 ± 0.1 a	0.39 ± 0.02 d	1.30 ± 0.04 ab	15.2 ± 0.4 c	25.1 ± 2.2 bc	6.2 ± 0.4 a	127.6 ± 3.2 c	499.8 ± 4.0 bc
50IN/50ON	8.5 ± 0.0 a	0.39 ± 0.01 cd	1.24 ± 0.04 bc	17.6 ± 0.1 b	28.4 ± 2.8 ab	6.6 ± 0.5 a	138.0 ± 5.3 b	465.5 ± 12.5 cd
25IN/75ON	8.5 ± 0.0 a	0.41 ± 0.00 c	1.21 ± 0.04 c	20.0 ± 0.1 a	30.8 ± 2.3 a	6.5 ± 1.0 a	166.8 ± 5.1 a	449.3 ± 18.9 d

EC electrical conductivity, SOC soil organic carbon, NO_3_^−^-N nitrate nitrogen, NH_4_^+^-N ammonium nitrogen, P phosphorus, K potassium.

**Fig 1:**
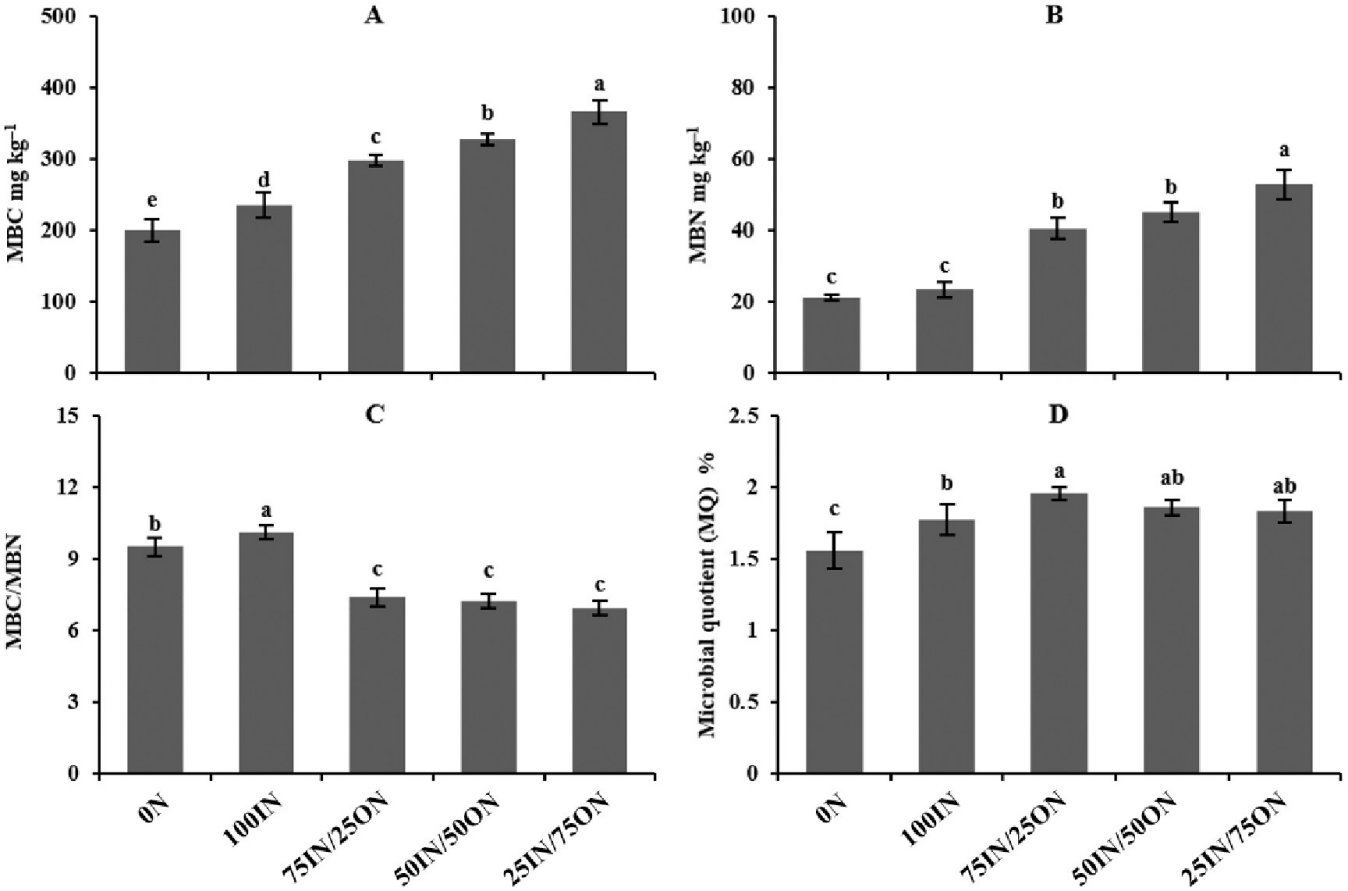
Changes in microbial biomass carbon (MBC) (A), microbial biomass nitrogen (MBN) (B), the ratio of MBC to MBN (C) and microbial quotient (MQ) (D) under different fertilization treatments after celery (the fifteenth-season) harvest in January 2017.

Manure-amended treatments significantly increased both soil MBC by 26.78%–82.91% and MBN by 73.94%–151.45% compared with chemical fertilization treatments (0N and 100IN) (Fig 1A and 1B). Moreover, these parameters increased as the manure application rate increased. The MBC/MBN ratios were highest in the 100IN treatment, followed by the 0N treatment, and the lowest values were observed in manure-amended treatments, while there were no significant differences among manure-amended treatments (Fig 1C). The microbial quotient (MQ) values in manure-amended soils were significantly higher than that in 0N treatment and slightly (*P* > 0.05) higher than that in 100IN treatment.

### 3.2 Changes in soil enzyme activities and geometric mean of enzyme activities

**Fig 2:**
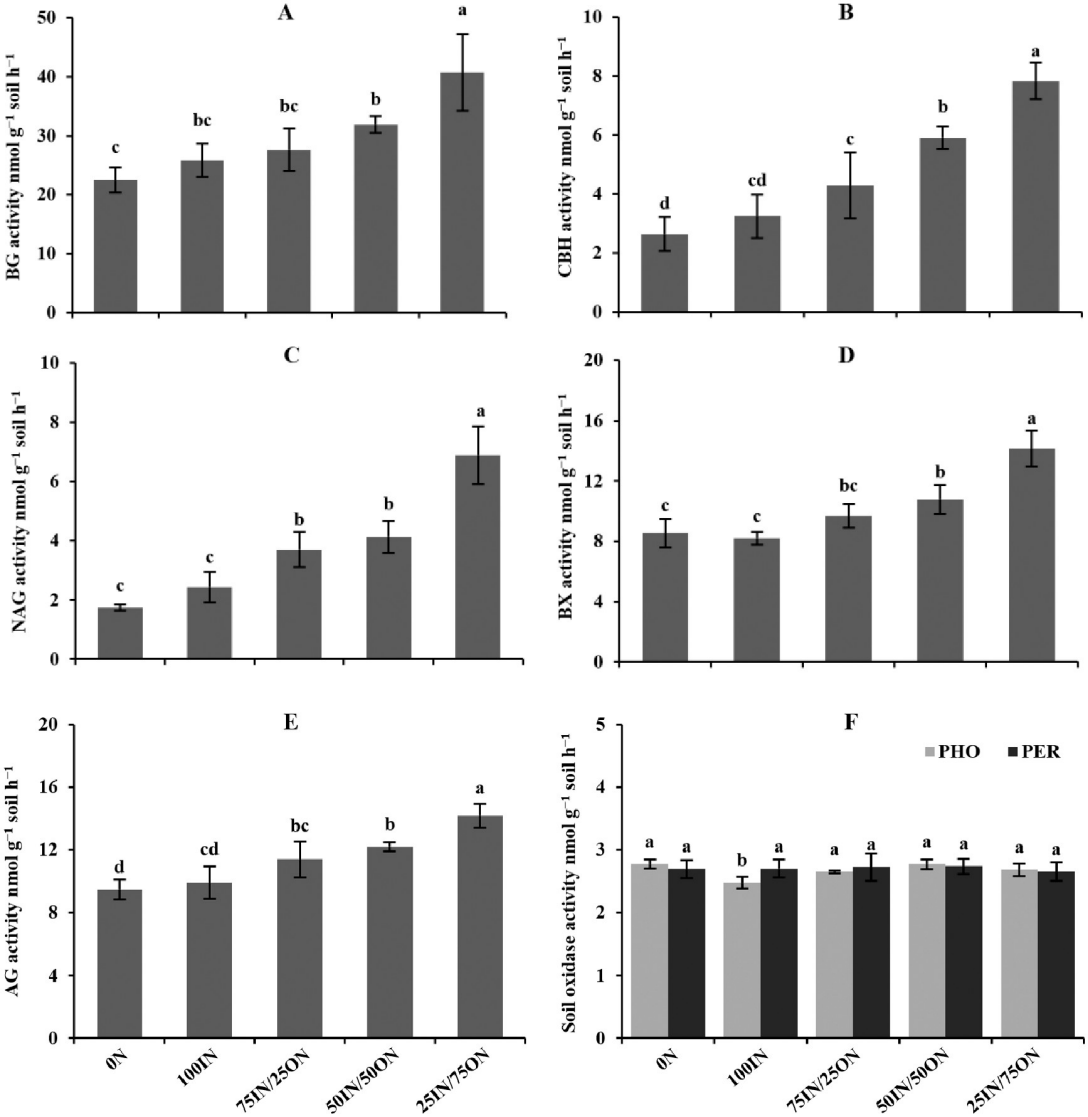
Changes in soil extracellular enzyme activities (A: BG activity; B: CBH activity; C: NAG activity; D: BX activity; E: AG activity and F: Soil ox idase activity) under different fertilization treatments. AG α-Glucosidase, BG β-Glucosidase, CBH β-Cellobiosidase, BX β-Xylosidase, NAG N-Acetyl-glucosaminidase, PHO Phenol oxidase, PER Peroxidase.

Manure substitution of chemical fertilizer significantly affected soil extracellular enzyme activities (EEAs) with the exception of peroxidase (PER) (Fig 2). Compared to chemical fertilization treatments, soil hydrolase activities were slightly higher under 75IN/25ON treatment, and significantly higher under 50IN/50ON and 25IN/75ON treatments by 23.4%−81.1% (BG), 82.3%−196.4% (CBH), 69.9%−296.0% (NAG), 31.3%−65.7% (BX), and 23.3%−49.5% (AG), respectively. Moreover, no significant differences were observed in soil hydrolase activities between 0N and 100IN treatments. Soil oxidase activities (phenol oxidase: PHO and PER) showed different trends compared with soil hydrolase activities. The PHO activity did not differ among manure-amended treatments and 0N treatment, but the PHO activity was significantly lower in 100IN treated soils than that in the manure-amended soils (by 7.0%–11.8%). Additionally, there were no significant differences in the PER activity among different fertilization treatments.

Compared with chemical fertilization treatments, the geometric mean of the enzyme activities under manure-amended treatments were significantly increased by 17.8%–75.2% (Gmea), 23.9%–121.3% (GH) and 19.2%–126.3% (GH/GOR), respectively (Table 5). The values of Gmea, GH and GH/GOR increased as the manure application rate increased. Additionally, no significant differences were observed in the GOR values among different fertilization treatments.

**Table 5:** Changes in the geometric mean of the assayed enzyme activities (Gmea), hydrolase (GH) and oxidase (GOR) under different fertilization treatments.

	Gmea	GH	GOR	GH/GOR
0N	4.83 ± 0.28 c	6.08 ± 0.53 c	2.73 ± 0.07 a	2.23 ± 0.23 d
100IN	5.24 ± 0.45 c	6.96 ± 0.80 c	2.59 ± 0.11 a	2.69 ± 0.30 cd
75IN/25ON	6.17 ± 0.55 b	8.62 ± 1.02 b	2.69 ± 0.12 a	3.20 ± 0.38 bc
50IN/50ON	6.94 ± 0.07 b	10.03 ± 0.14 b	2.76 ± 0.09 a	3.64 ± 0.15 b
25IN/75ON	8.47 ± 0.65 a	13.45 ± 1.37 a	2.67 ± 0.07 a	5.04 ± 0.44 a

Data are means ± S.E., n = 3. Different lowercase letters indicate significant differences among different treatments at the *P* < 0.05 level.

**Fig 3:**
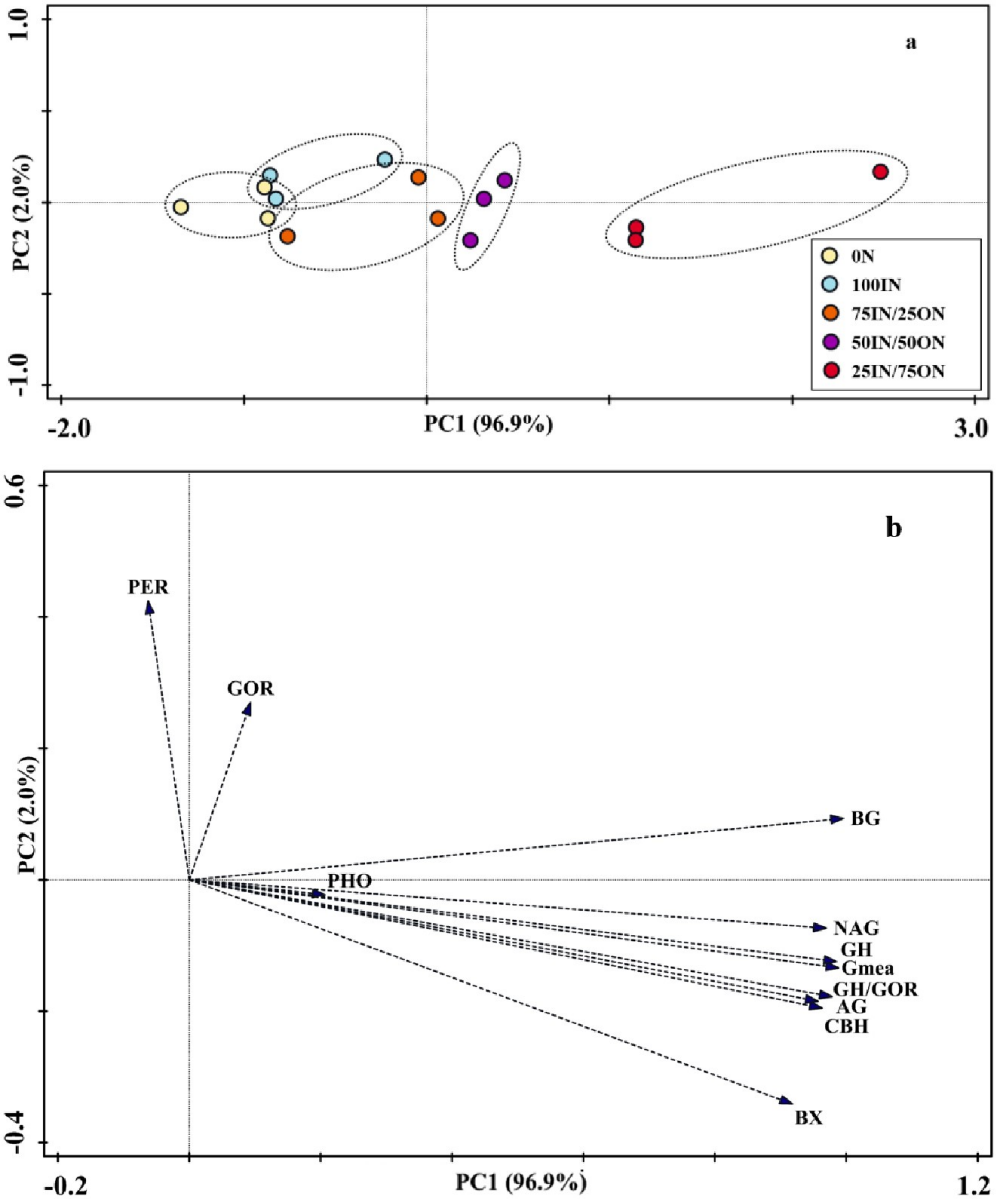
Principal component analysis (PCA) of soil extracellular enzyme activity profiles (a), and loading values for individual soil extracellular enzyme activities (b) from different fertilizer treatments.

Results from principal component analysis (PCA) indicated that soil enzyme activities changed under different fertilization treatments (Fig 3). The first principal component (PC1) explained 96.9%, and the second (PC2) explained 2.0%, of the total variance (Fig 3a). Soil enzyme activity profiles showed significant separation among the five fertilization treatments. Along with PC1, higher manure-amended treatments (50IN/50ON and 25IN/75ON) had higher scores than chemical fertilization treatments. Along PC2, soil enzyme activities profile scores did not differ among the five treatments. The PC loadings for soil enzyme activities (Fig 3b) and PC scores indicated that higher manure-amended treatments enhanced soil hydrolase (BG, CBH, NAG, BX and AG) activities, and the values of Gmea, GH and GH/GOR.

### 3.3 Changes in soil microbial community structure

**Fig 4:**
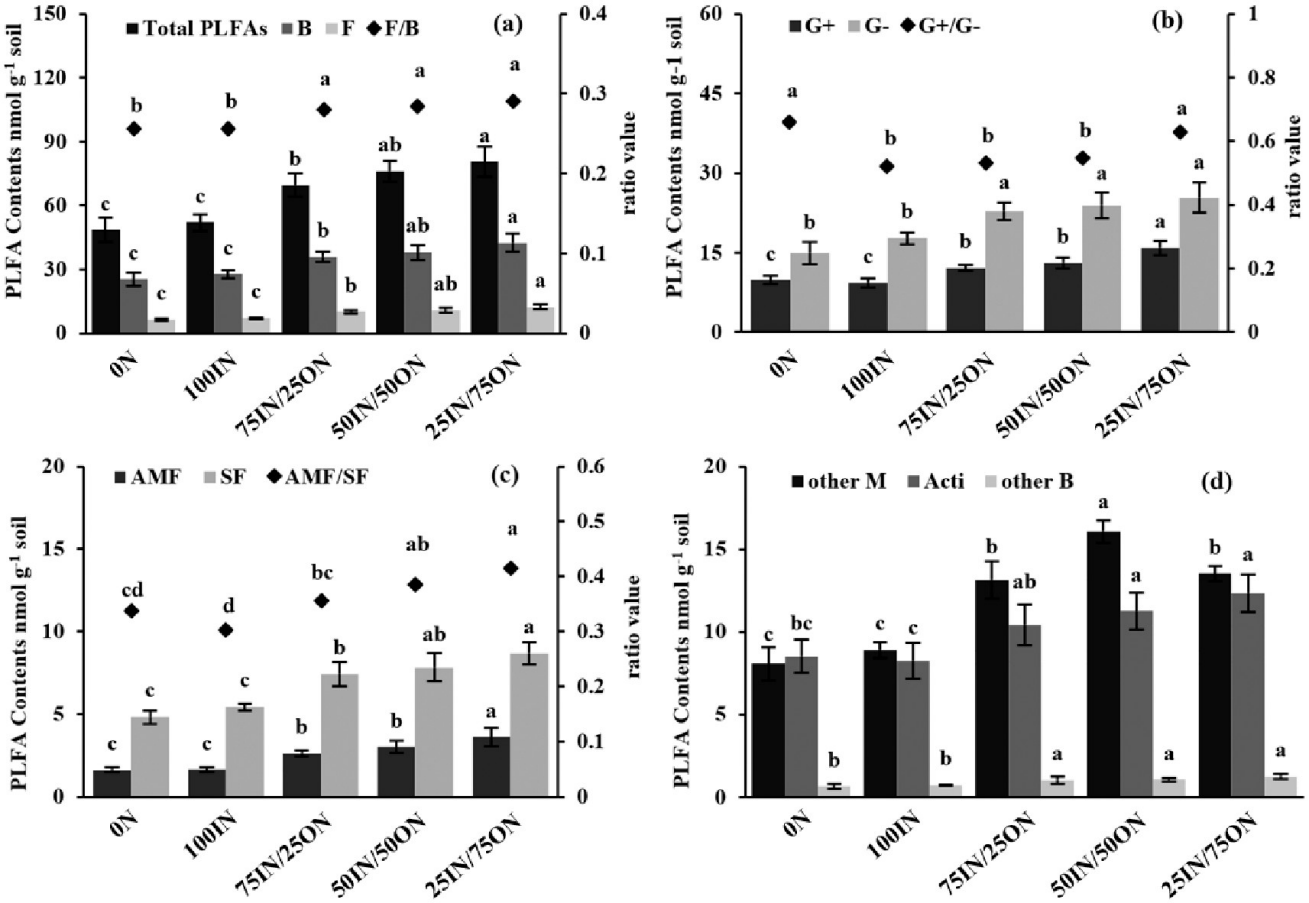
Changes in concentrations (nmol g^−1^ soil) of total PLFAs and microbial subgroups of PLFAs and associated ratios under different fertilization treatments. B: bacteria, F: fungi, G+: Gram-positive bacteria; G−: Gram-negative bacteria; AMF: arbuscular mycorrhizal fungi, SF: saprotrophic fungi, M: microorganism, Acti: actinomycetes.

After celery harvest, total PLFA contents in manure-amended treatments (69.55–80.63 nmol g^−1^ soil) were significantly higher than those in chemical fertilization treatment (48.46–51.98 nmol g^−1^ soil), and total PLFA contents increased as the manure application rate increased (Fig 4a). No significant differences were found in total PLFA contents between 0N and 100IN treatments. Similar trends were observed for the contents of fungi (saprotrophic fungi (SF) and arbuscular mycorrhizal fungi (AMF)), bacteria (Gram-positive (G+) bacteria and Gram-negative (G−) bacteria) and actinomycetes. Specific trends were as follows: 25IN/75ON > 50IN/50ON > 75IN/25ON > 100IN and 0N (Fig 4a, 4b, 4c and 4d).

The ratios of fungi to bacteria (F/B) and arbuscular mycorrhizal fungi to saprotrophic fungi (AMF/SF) in manure-amended treatments were significantly increased by 9.51%−13.48% and 5.55%−37.09%, respectively, compared to those in chemical fertilization treatments (Fig 4a and 4c). Compared to 100IN treatment, the ratio of Gram-positive/Gram-negative bacteria (G+/G−) slightly increased under 75IN/25ON and 50IN/50ON, and significantly increased by 20.26% under 25IN/75ON. Additionally, the G+/G− values were highest in the 0N treatment (0.66) and significantly higher by 26.33% than 100IN treatment (Fig 4b).

**Fig 5:**
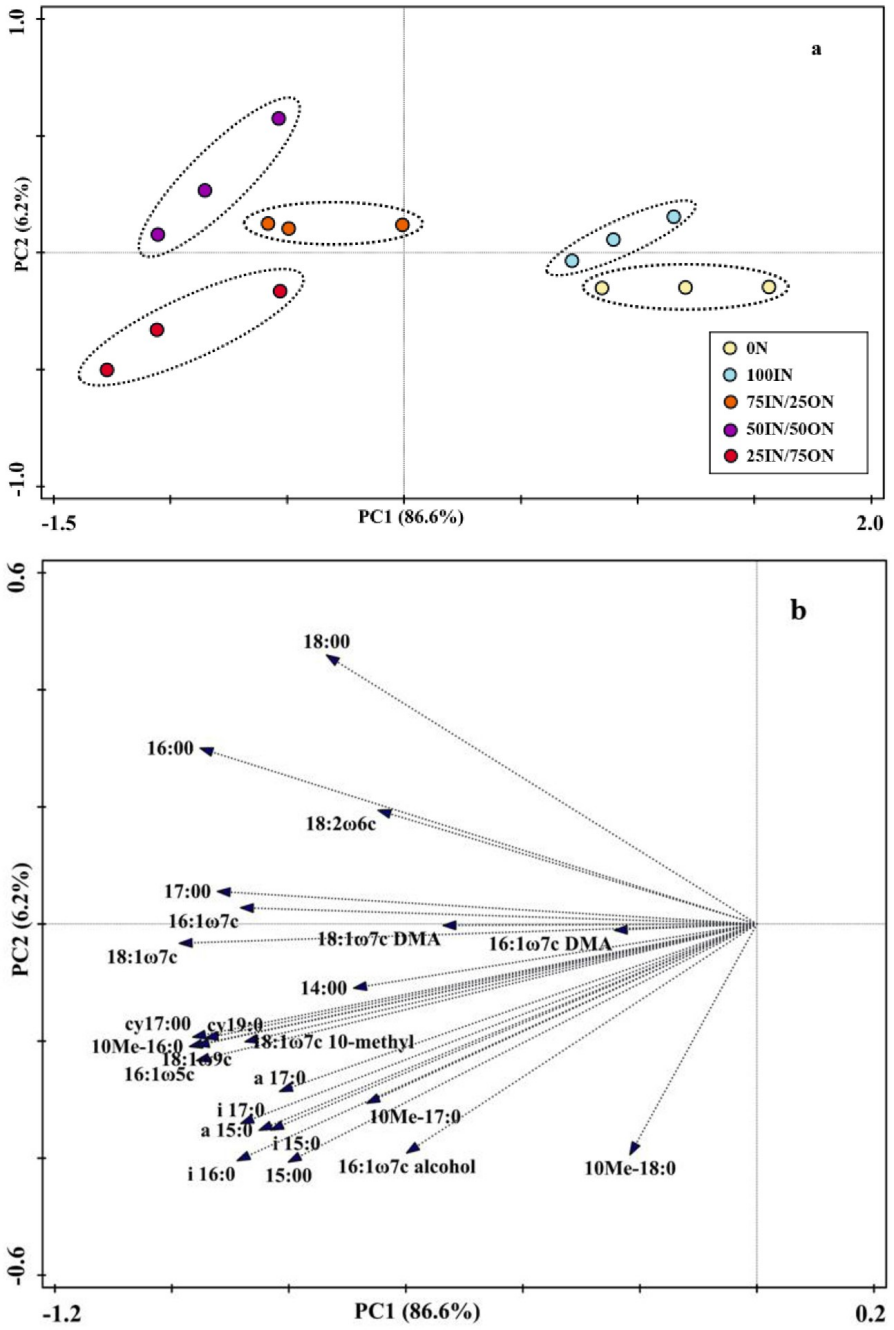
Principal component analysis (PCA) of soil phospholipid fatty acids (PLFAs) (a), and loading values for individual PLFAs (b) from different fertilizer treatments.

The PCA of the 24 PLFA data indicated that soil microbial community structure was markedly affected by manure application, but was not different (*P* > 0.05) between 0N and 100IN treatments, indicated by their closest scores along the first principal component (PC1) and the second component (PC2) (Fig 5a). The first two components, PC1 and PC2 explained 86.6% and 6.2% of the total variance in PLFA profiles (Fig 5). PC1 axis differentiated manure-amended treatments from chemical fertilization treatments, whereas the PC2 axis did not differentiate fertilization treatments. The PC loadings for individual PLFA (Fig 5b) and PC scores indicated that manure addition enhanced the vast majority of PLFAs contents.

### 3.4 Changes in soil microbial community diversity and bacterial physiological stress indices

Soil microbial community and functional (enzyme) diversity in this study varied among different fertilization treatments (Table 6). Compared to chemical fertilization treatments, Shannon–Wiener diversity index (H′_M_) and Margalef richness index (SR) slightly increased under 75IN/25ON treatment, significantly increased under 50IN/50ON and 25IN/75ON treatment by 1.44%–3.74% and 10.42%–17.15%, respectively. Contrary to H′_M_ and SR, higher rates of manure input (50IN/50ON and 25IN/75ON) significantly reduced the Pielou evenness index (J) by 2.58%–6.10% compared to chemical fertilization treatments. Soil enzyme function diversity index (H′_E_) calculated from all enzyme activities showed a similar tendency as H′_M_: 25IN/75ON > 50IN/50ON > 75IN/25ON > 100IN > 0N.

**Table 6:** Changes in soil microbial PLFA and enzyme diversity under different fertilization treatments. Data are means ± S.E., n = 3. Different lowercase letters indicate significant differences among different treatments at the *P* < 0.05 level.

Treatment	Soil enzyme diversity	Microbial PLFA diversity		
	H′_E_	H′_M_	SR	J
0N	1.84 ± 0.02 d	3.14 ± 0.01 c	10.39 ± 0.10 b	0.84 ± 0.01 a
100IN	1.88 ± 0.02 c	3.14 ± 0.02 c	9.91 ± 0.40 b	0.83 ± 0.01 b
75IN/25ON	1.91 ± 0.02 b	3.15 ± 0.03 c	10.40 ± 0.39 b	0.81 ± 0.01 bc
50IN/50ON	1.93 ± 0.00 ab	3.19 ± 0.02 b	11.61 ± 0.12 a	0.79 ± 0.01 d
25IN/75ON	1.94 ± 0.00 a	3.26 ± 0.00 a	11.47 ± 0.57 a	0.80 ± 0.01 cd

H′_E_: Shannon’s diversity index (enzyme), H′_M_:Shannon’s diversity index (PLFAs), SR: Margalef richness index, J: Pielou evenness index

**Fig 6:**
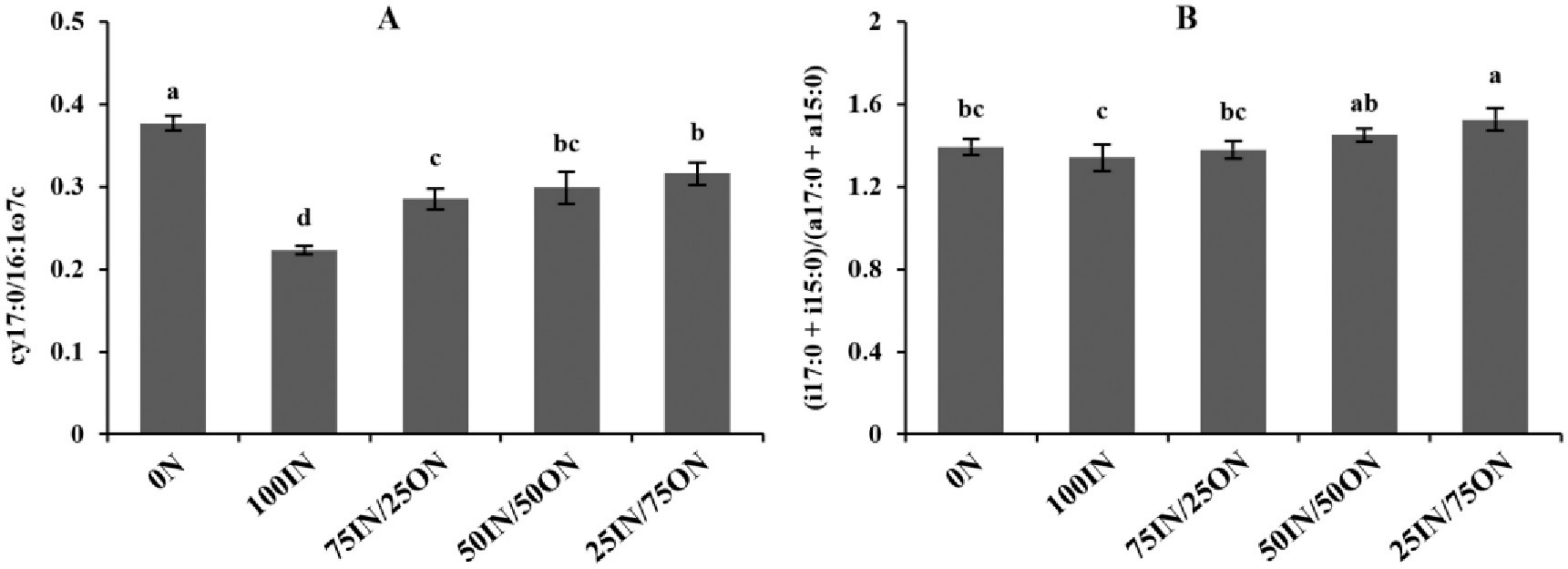
Changes in soil bacterial physiological stress indices (A: Cy17:0/16:1ω7c; B: (i17:0 + i15:0)/(a17:0 + a15:0)) under different fertilization treatments.

The cy17:0/16:1ω7c and (i17:0 + i15:0)/(a17:0 + a15:0) values, which indicated bacterial physiological stress, varied from 0.223 to 0.377 and from 1.34 to 1.53, respectively. The cy17:0/16:1ω7c values were highest in the 0N treatment, followed by manure-amended treatments, and the lowest value was observed in the 100IN treatment (Fig 6). Compared to the 100IN treatment, the (i17:0 + i15:0)/(a17:0 + a15:0) values slightly increased under the 75IN/25ON treatment, significantly increased by 8.16% under the 50IN/50ON treatment and by 13.89% under the 25IN/75ON treatment.

### 3.5 Correlations between soil physicochemical properties and soil microbial characteristics

**Fig 7:**
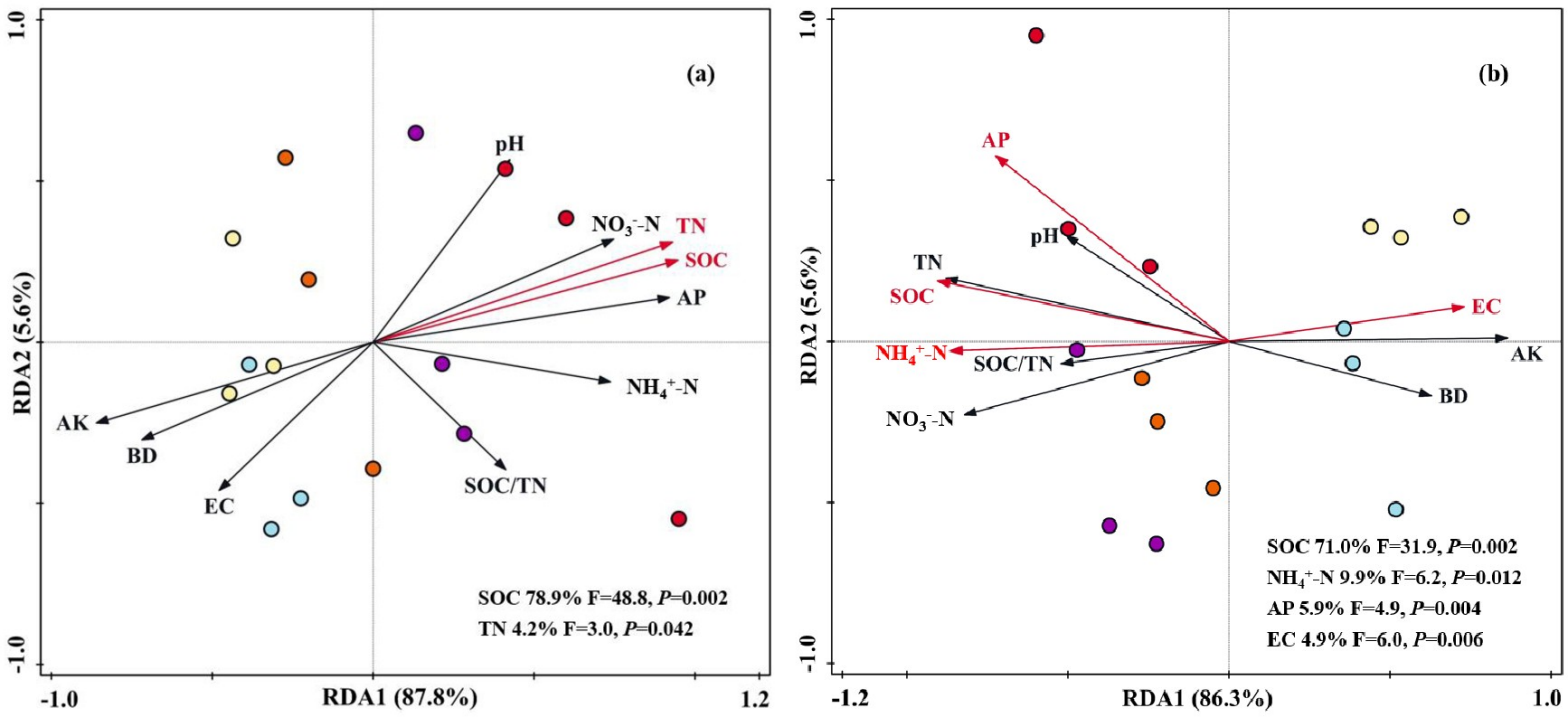
Redundancy analysis (RDA) of soil enzyme activities (a), microbial community structure (b) constrained by soil physicochemical properties under different fertilization treatments.

According to the Redundancy analysis (RDA) of the activities of all enzymes constrained by soil physicochemical properties, the first and second ordination RDA axis (RDA1 and RDA2) explained 87.8% and 5.6% of the total variance, respectively (Fig 7a). The RDA confirmed that SOC content (F = 48.8, *P* = 0.002) and TN content (F = 3.0, *P* = 0.042) were strongly associated with soil enzyme activities and explained 78.9% and 4.2% of the total variance of enzyme activities data, respectively (Fig 7a). Another RDA was performed using soil physicochemical properties as explanatory variables and soil PLFAs profiles as response variables (Fig 7b). RDA1 and RDA2 accounted for 86.3% and 5.6% of the total variance of the PLFA data, respectively. Forward selection indicated that SOC content (F = 31.9, *P* = 0.002) was the most important variable, explaining 71.0% of the total variance of the PLFA data, followed by NH_4_^+^-N content (9.9%, F = 6.2, *P* = 0.012), available P content (5.9%, F = 4.9, *P* = 0.004) and soil EC (4.9%, F = 6.0, *P* = 0.006).

## 4. Discussion

### 4.1 Effects of manure substitution of chemical fertilizer on soil physicochemical properties

Organic amendments and substitution of inorganic fertilizers are increasingly recommended as an important means to sustain crop yield and soil quality [19]. Our results indicated that organic fertilizer addition improved soil nutrient-related properties by increasing SOC, NO_3_^−^-N, NH_4_^+^-N and available P contents compared to inorganic fertilizer amendments (Table 4). These results were in agreement with those from other vegetable fields [37]. We suggest two main explanations for these results in this GVP system. First, the additions of manure increased the duration for C accumulation and built up soil C pools [38]. Secondly, Jiao et al. [39] indicated that manure application could improve the nutrient retention ability of soil.

Additionally, we found that manure addition decreased soil available K content compared to inorganic fertilizer additions, which is in contrast to other studies, which showed annual addition of manure increased soil available K content in double maize and maize–wheat double-cropping systems [40, 41], but some researchers studying GVP system indicated that manure application markedly decreased soil available K content [42]. Chen et al. [43] and Ni et al. [44] indicated that when the vegetables are harvested, a large amount of K is removed from the soil. In other words, soil K availability was reduced in manure-amended plots due to vegetable uptake, driven by increased vegetable growth (S1 Table).

Soil pH and EC values can directly or indirectly affect the nutrient availability of soil, thus playing vital roles in soil fertility and quality [45]. We found that manure application increased soil pH values by about 2.5%, while reduced soil EC values by 16.7%–21.1%, compared to 100IN treatment. These results were supported by the findings of Wei et al. [6], who found that manure inputs alleviated the negative effect of chemical fertilizer application on soil pH. The possible explanation was that organic manure inputs could alleviate nitrification activities (acid-producing process) caused by chemical N inputs [46]. Mokolobate and Haynes [47] indicated organic manure contains humic-type substances with large amounts of carboxyl, phenolic, and enolic functional groups, which can consume the protons (e.g., Al^3+^, H^+^, etc.), which perhaps explained the reduction of soil EC values in manure-amended soils.

### 4.2 Effects of manure substitution of chemical fertilizer on soil enzyme activity

Soil extracellular enzyme activities (EEAs) are vital indicators of soil microbial activity, and they are closely associated with SOC decomposition and nutrient cycling [48]. Based on their functions, soil enzymes can be divided into hydrolases and oxidases that decompose substrates of various composition and complexity, which are strongly affected by fertilization [49]. It’s worth noting that the positive effects of manure application on soil hydrolase activities were supported by most scholars [7, 42], but the influences of manure application on soil oxidase activities were controversial [50, 51]. In our study, manure application was beneficial to enhance soil hydrolase (BG, CBH, NAG, BX, and AG) activities (Fig 2). The trends in soil hydrolase activities could be partially interpreted by soil characteristics related to carbon and nutrient availability, which are well known to be strongly influenced by fertilizer management practices [20, 50]. Moreover, manure application could stimulate the growth of soil microorganisms [41], thus increasing soil hydrolase activities.

Soil oxidases are generally produced by fungi, and their activities were different from soil hydrolase activities in soil [50, 52]. In our study, soil PHO and PER activities were not affected by manure application, whereas NPK chemical fertilizer application (100IN) markedly inhibited soil PHO activity relative to other treatments. Similar results have been reported in other studies [53–55], which also found that chemical N inputs were shown to depress soil oxidase (PHO) activity. Sinsabaugh [52] found that oxidase (PER and PHO) activities generally increase with soil pH. In our study, chemical N input could reduce soil pH, and then decrease oxidase activities. These studies indicated that manure application mainly stimulated the C-hydrolase activities, which indicated a high C turnover rate in manure-amended soils.

### 4.3 Effects of manure substitution of chemical fertilizer on soil microbial community composition

Soil microorganisms can be used as pivotal indicators of biogeochemical cycles because of their diversity, functional traits, dispersal ability and density [56]. PLFA analyses can provide information on several levels (“metrics”) of the soil microbial community, from a whole-community profile to specific group abundance, microbial diversity, and bacterial physiological stress (Figs 4 and 6 and Table 6) [57]. Recently, several studies have investigated the response of soil community structure and abundance to manure application in different ecosystems. In our study, the contents of all and individual microbial groups (e.g., fungi, bacteria, and actinomycetes, etc.) and microbial biomass (MBC and MBN) increased with increasing manure input rates, which was in accordance with the findings of Yue et al. [41]. These findings were mainly attributed to the adequate supply of C and nutrient resources by manure application, which was beneficial for microbial growth and reproduction [58]. Additionally, the microorganisms present in organic manure (S2 Table) could also be contributing to the enhancement of soil microbial biomass [59].

Manure application not only increased all individual microbial groups’ biomass, but also changed the microbial community structure (Fig 4). In line with the results of previous research on agricultural soil [60], we found that the F/B, G+/G− and AMF/SF ratios increased after 8 years of increased manure input (Fig 4). These changes indicate that some species (fungi, G+ bacteria, and AMF) adapt better to manure application than other species (G− bacteria and SF). According to previous studies, the utilization of exogenous organic resources (e.g., manure) by microorganisms could result in changes to community composition: fast-growing G− bacteria proliferate soon after organic materials addition, and then their population size decreases, promoting the growth of other more slowly-growing microorganisms such as G+ bacteria or fungi [61]. Additionally, manure application could promote soil aggregation and create more large pores, which facilitate fungal growth [62]. These studies perhaps explained the increase of G+/G− and F/B ratios in manure-amended soils. Previous studies have concluded that different microorganisms have different effects on nutrient cycling and SOC accumulation in soil and that higher F/B, G+/G− and AMF/SF ratios are favorable for SOC accumulation and soil aggregation [63, 64]. Therefore, we speculate that manure application optimized soil microbial community structure and could be beneficial for soil fertility and biological quality.

### 4.4 Effects of manure substitution of chemical fertilizer on soil microbial community diversity and physiological stress

Soil microbial community diversity and physiological stress were affected by manure application (Table 6 and Fig 6). Ge et al. [65] and Yu et al. [66] found that the values of H′_M_ and SR were increased upon manure inputs in different agricultural soils of Northern China. Similarly, our results indicated that manure application not only increased soil microbial community diversity (H′_M_) and richness (SR) but also reduced soil microbial community evenness (Table 6). It has been suggested that manure application could (1) improve soil structure and (2) increase C and nutrient resource diversity, and subsequently improve soil microbial community diversity and richness [58, 67]. Interestingly, different microorganisms have been reported to be differently affected by fertilization, owing to their diverse structure and physiology (e.g., manure addition was more favorable to the growth of some species, such as fungi, etc.) [68, 69]. This could be the reason why soil microbial community evenness decreased in manure-amended soils.

The cy17:0/16:1ω7c and (i17:0 + i15:0)/(a17:0 + a15:0) ratios were denoted as bacterial physiological stress indices, which indicated bacterial physiological status in response to environmental stress [69]. Higher values of these indices are associated with decreased bacterial growth rate and increased SOC and nutrient limitation [33]. In our study, the higher values of bacterial stress indices suggested that nutritional or environmental stress was more limiting to the growth of bacteria in the manure-amended soils. This is in contrast to the usual expectation that the addition of organic fertilizers would decrease the values of these indices [69], but some studies do support our conclusions [70]. Our results could be caused by competition between fungi and bacteria for organic substrate and nutrients [71]. Namely, the rapid and massive proliferation of fungi after the addition of manure had antagonistic effects on bacterial growth.

### 4.5 Linking soil microbial community composition and enzyme activity to soil physicochemical properties

Understanding the main environmental drivers that regulate microbial communities and their functions is an important challenge in the field of microbial ecology [6]. The RDA results (Fig 7a) indicated that the majority of the variation in soil enzyme activities could be explained by soil nutrient characteristics such as SOC contents (78.9%) and TN contents (4.2%). In agreement with our results, Wang et al. [28] found that the changes in soil enzyme activities were strongly related to soil total C and N contents in tropical forests. The reason maybe that sufficient C and N resources (derived from manure) could alleviate C and N limitations of soil microbial metabolism, and thus enhance the secretion of enzymes [72].

It has been found in many studies that environmental factors and soil management practices strongly affect soil microbial structure and composition [73]. The RDA results (Fig 7b) indicated that SOC content (71.0%), NH_4_^+^-N content (9.9%), available P content (5.9%) and soil EC (4.9%) play important roles in shaping soil microbial community composition. This means that manure addition results in lower soil EC and improved soil chemical properties (SOC, NH_4_^+^-N and available P), thus inducing a strong response to the changes in soil microbial structure. Luo et al. [60] and Ma et al. [74] also reported that C and nutrients (N and P) supplied by manure were major factors affecting soil microbial community structure in semiarid farmland and subtropical paddy soils, respectively. In conclusion, the changes in soil microbial characteristics were driven by changes in soil physicochemical properties caused by different fertilization patterns: the ten selected environmental factors together accounted for >90% of the total variation (Fig 7a and 7b), and SOC and available nutrient (N) contents, rather than other soil physicochemical properties, are the vital drivers among them.

## 5. Conclusions

The 8-year field experiment revealed the responses of soil physicochemical properties, soil enzyme activities, and microbial community structure to different fertilization treatments in a GVP of Tianjin, China. Our results suggested that manure substitution is beneficial for improving soil quality (i.e., increase in SOC and nutrient contents) and potential crop productivity. Moreover, we confirmed that manure substitution of chemical fertilizer, especially manure application at higher rates, significantly affected soil microbial characteristics in four aspects: (1) increased in microbial biomass and diversity, (2) shifted in community composition (increased F/B, AMF/SF, and G+/G− ratios), (3) increased in bacterial physiological stress, and (4) enhanced in microbial hydrolytic activity. We further found that SOC content and nutrient (N)-related properties (especially SOC) played important roles in determining the effects of different fertilization treatments on the changes in soil microbial characteristics. In conclusion, the practices of organic manure substitution of chemical fertilizer, especially at high manure application rates (20.8–31.2 Mg ha^−1^ yr^−1^), could be a promising approach to develop sustainable agriculture and was beneficial for vegetable development and soil quality in GVP systems.

## Supporting information

S1 Table Effect of 8 years of different fertilization treatments on vegetable yields (Mg ha^−1^) in a solar-greenhouse system. (DOCX)

S2 Table The concentrations (nmol g^−1^ fertilizer) of total PLFAs and microbial subgroups of PLFAs in the organic manure used in our study. (DOCX)

S1 Data (XLSX)
