# Supplementary material for: Substitution of manure for chemical fertilizer affects soil microbial community diversity, structure and function in greenhouse vegetable production systems
Source: PLoS One. 2020 Feb 21;15(2):e0214041. doi: 10.1371/journal.pone.0214041 (PMC7034837; doi:10.1371/journal.pone.0214041)
Supplement: S1 Table — (DOCX) [file pone.0214041.s001.docx]

Table S1 Effect of 8 years of different fertilization treatments on vegetable yields (Mg ha^−1^) in a solar-greenhouse system.

| Year | Crop | Treatments | | | | |
| --- | --- | --- | --- | --- | --- | --- |
|  |  | 0N | 100IN | 75IN/25ON | 50IN/50ON | 25IN/75ON |
| 2009 | Celery | 116.70±0.70c | 119.57±2.49ab | 121.77±1.42a | 119.67±0.51ab | 119.8±1.85a |
| 2010 | Tomato | 88.45±1.21c | 95.43±2.44b | 100.61±1.41a | 98.43±1.16ab | 95.83±1.85b |
|  | Celery | 137.97±2.79c | 143.99±0.61b | 151.4±2.28a | 145.03±0.78b | 146.54±1.04b |
| 2011 | Tomato | 99.02±0.61c | 107.41±1.25b | 114.76±1.05a | 108.97±1.39b | 106.95±1.25b |
|  | Celery | 109.49±1.78c | 124.07±0.88b | 133.1±1.64a | 125.46±6.6b | 122.57±3.76b |
| 2012 | Tomato | 71.93±1.33c | 91.77±3.42b | 100.66±0.93a | 97.27±0.88a | 99.55±0.71a |
|  | Celery | 112.38±1.22c | 123.61±1.84b | 126.27±1.78ab | 127.66±0.87ab | 129.98±3.52a |
| 2013 | Tomato | 76.25±2.25e | 83.43±0.81d | 87.29±0.66c | 95.58±1.42b | 102.38±2.04a |
|  | Celery | 93.24±3.37c | 103.47±1.84b | 106.47±1.93ab | 107.53±1.78ab | 109.67±2.42a |
| 2014 | Tomato | 75.58±2.78d | 86.76±1.89c | 90.6±1.05bc | 94.65±3.73ab | 99.58±4.37a |
|  | Celery | 88.7±3.08c | 96.58±2.93b | 99.65±1.84b | 101.5±2.89b | 108.71±2.23a |
| 2015 | Tomato | 73.91±3.56d | 85.09±1.12c | 90.6±1.05bc | 94.32±5.69ab | 101.25±6.62a |
|  | Celery | 92.25±1.56c | 92.11±1.99c | 94.37±2.05bc | 96.41±2.2ab | 99.19±1.4a |
| 2016 | Tomato | 62.98±2.56c | 73.83±8.92bc | 78.63±8.95ab | 82.45±7.22ab | 90.16±4.61a |
|  | Celery | 84.09±5.37c | 92.38±4.25bc | 97.18±5.56b | 101.04±3.35ab | 106.87±5.6a |
