# Supplementary material for: Substitution of manure for chemical fertilizer affects soil microbial community diversity, structure and function in greenhouse vegetable production systems
Source: PLoS One. 2020 Feb 21;15(2):e0214041. doi: 10.1371/journal.pone.0214041 (PMC7034837; doi:10.1371/journal.pone.0214041)
Supplement: S2 Table — (DOCX) [file pone.0214041.s002.docx]

Table S2 The concentrations (nmol g^−1^ fertilizer) of total PLFAs and microbial subgroups of PLFAs in the organic manure used in our study.

|  | Total PLFAs | Bacteria | Fungi | Actinomycetes | AMF | SF | G+ | G− |
| --- | --- | --- | --- | --- | --- | --- | --- | --- |
| Organic manure | 12.5 | 6.7 | 1.4 | 1.5 | 0.05 | 1.3 | 4.2 | 2.1 |

Note: G+: Gram-positive bacteria; G−: Gram-negative bacteria; AMF: arbuscular mycorrhizal fungi, SF: saprotrophic fungi.
